# Supplementary material for: Modulation of Extracellular Matrix Composition and Chronic Inflammation with Pirfenidone Promotes Scar Reduction in Retinal Wound Repair
Source: Cells. 2024 Jan 16;13(2):164. doi: 10.3390/cells13020164 (PMC10814251; doi:10.3390/cells13020164)
Supplement: Supplementary file 1 [file cells-13-00164-s001.zip › cells-2795332-supplementary.pdf]

Supplementary materials

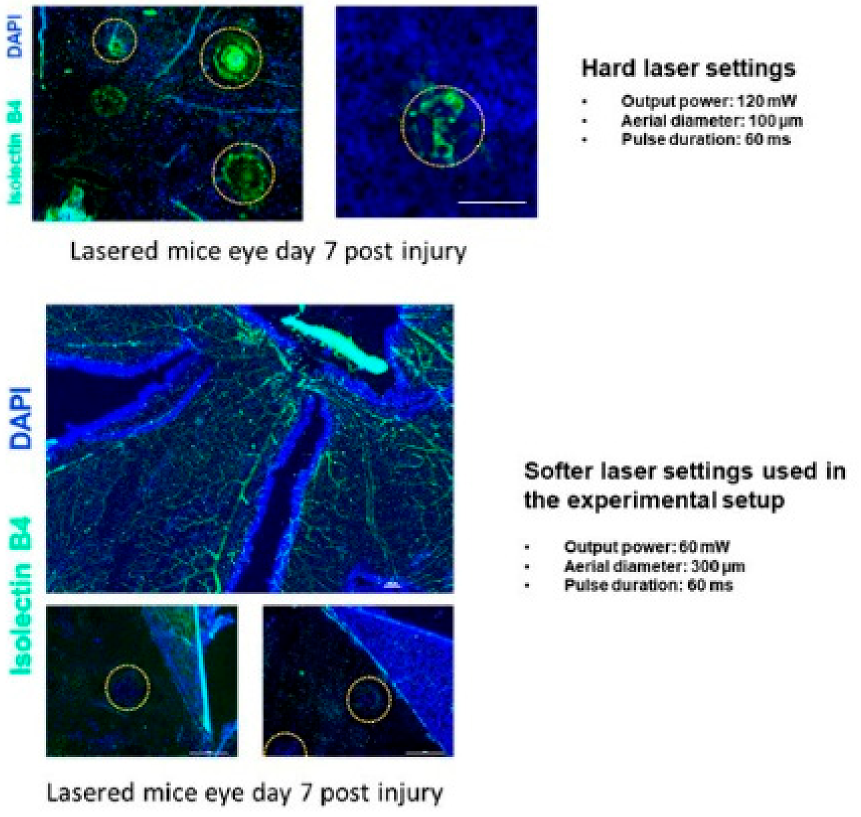

Supplementary Figure S1: Isolectin GS-B4 staining for CNV detection without Bruch's membrane rupture after modification of the laser setting. The dotted circles denote the area of the laser spot. The scale bar represents 100 μm.

Supplementary Table S1: Fold change of ECM component proteins post lasered samples (significant changes in bold)

	Day 7		D21		D35		D49	
**Gene Symbol**	**Fold Change**	**P-Value**	**Fold Change**	**P-Value**	**Fold Change**	**P-Value**	**Fold Change**	**P-Value**
Col1a1	2.93	0.26	0.41	0.40	1.02	0.55	1.21	0.73
Col2a1	1.68	0.52	0.91	0.90	1.96	0.31	2.31	0.18
Col3a1	1.91	0.28	0.4	0.13	0.32	0.07	0.35	0.07
Col4a1	0.58	0.12	1.11	0.62	0.72	0.17	0.74	0.21
Col4a2	0.99	0.80	0.77	0.88	1.04	0.81	1.28	0.81
Col4a3	2.54	0.08	1.5	0.34	**3.15**	0.00	**4.32**	0.01

Col5a1	2.26	0.41	0.87	0.93	2.16	0.44	2.85	0.17
Col6a1	1.46	0.92	0.56	0.51	1.33	0.86	1.69	0.77
Ecm1	1.84	0.49	0.94	0.87	1.81	0.49	2.42	0.18
Emilin1	2.02	0.55	0.89	0.90	1.72	0.82	2.23	0.41
Fbln1	4.89	0.22	5.45	0.13	**12.38**	0.00	**4.35**	0.00
Fn1	1.53	0.81	0.6	0.55	1.36	0.94	1.82	0.58
Hapln1	1.29	0.66	0.7	0.41	1.12	0.96	1.31	0.65
Lama1	1.72	0.67	0.8	0.65	1.56	0.85	2.15	0.37
Lama2	1.27	0.51	0.98	0.92	0.91	0.67	1.06	0.95
Lama3	1.42	0.46	1.03	0.78	1.27	0.64	1.74	0.18
Lamb2	1.71	0.39	1.08	0.98	1.36	0.74	1.59	0.47
Lamb3	1.26	0.89	0.7	0.44	1.11	0.84	1.67	0.49
Lamc1	2.34	0.66	0.62	0.67	2.44	0.62	3.47	0.24
Sparc	1.46	0.82	0.88	0.77	1.11	0.72	1.27	0.92
Spock1	1.38	0.91	0.73	0.64	1.54	0.78	1.87	0.49
Spp1	1.08	0.81	0.85	0.56	1.11	0.78	1.16	0.65
Syt1	1.52	0.63	0.6	0.54	1.5	0.67	1.95	0.29
Tnc	1.98	0.72	0.67	0.66	1.64	1.00	2.56	0.38
Vcan	2.09	0.26	2.03	0.22	**3.76**	0.00	**2.34**	0.03
Vtn	1.56	0.51	0.78	0.72	1.52	0.54	2.03	0.17

Supplementary Table S2: Transmembrane and adhesion molecules fold change laser treated samples (significant changes in bold)

	**Day 7**		**D21**		**D35**		**D49**	
**Gene Symbol**	**Fold Change**	**P-Value**	**Fold Change**	**P-Value**	**Fold Change**	**P-Value**	**Fold Change**	**P-Value**
Cd44	1.75	0.30	1.18	0.63	1.79	0.15	1.98	0.11
Cdh1	1.51	0.35	2.33	0.36	1.61	0.23	1.84	0.15

Cdh2	2.96	0.65	0.66	0.63	2.99	0.68	3.84	0.35
Cdh3	**0.68**	0.03	0.81	0.23	**0.65**	0.01	1.03	0.77
Cdh4	1.82	0.70	0.6	0.66	1.91	0.65	2.59	0.25
Cntn1	1.29	0.61	0.63	0.48	1.29	0.59	1.5	0.29
Ctnna1	1.66	0.54	0.75	0.76	1.59	0.61	1.83	0.39
Ctnna2	1.34	0.76	0.65	0.59	1.27	0.89	1.6	0.47
Ctnnb1	1.52	0.55	0.72	0.60	1.36	0.75	1.73	0.35
Icam1	**3.01**	0.05	1.35	0.56	2.53	0.09	3.59	0.06
Itga2	1.34	0.47	0.62	0.54	1.06	0.99	1.64	0.12
Itga3	1.13	0.81	0.74	0.60	0.95	0.89	1.44	0.36
Itga4	1.29	0.51	0.71	0.40	1.06	0.93	1.56	0.12
Itga5	2.04	0.66	0.7	0.70	2.04	0.68	3.06	0.24
Itgae	1.54	0.53	1.47	0.60	1.58	0.48	2.36	0.07
Itgal	2.08	0.11	1.2	0.69	1.89	0.08	**3.69**	0.03
Itgam	2.01	0.24	0.82	0.86	1.35	0.59	1.71	0.26
Itgav	1.23	0.60	0.67	0.50	1.27	0.47	1.59	0.15
Itgax	1.27	0.46	1.57	0.23	**0.65**	0.01	1.28	0.27
Itgb1	1.36	0.63	0.69	0.65	1.3	0.74	1.52	0.45
Itgb2	2.34	0.16	0.84	0.95	1.84	0.15	2.11	0.11
Itgb3	1.43	0.76	0.65	0.65	0.93	0.56	1.65	0.52
Itgb4	2.07	0.68	0.73	0.65	2.2	0.59	2.83	0.28
Ncam1	1.91	0.50	0.73	0.71	1.94	0.47	2.53	0.19
Ncam2	1.08	0.87	0.57	0.36	1.07	1.00	1.28	0.54
Pecam1	1.63	0.56	0.54	0.41	1.38	0.86	2	0.29
Postn	1.48	0.47	0.54	0.29	0.8	0.45	1.01	0.82
Sele	0.95	0.98	1.15	0.39	**0.59**	0.00	1.06	0.48
Sell	**0.7**	0.01	0.88	0.68	**0.62**	0.00	1.15	0.52

Selp	1.5	0.29	1.14	0.30	1.13	0.34	**2.23**	0.03
Sgce	1.27	0.71	0.8	0.69	1.35	0.64	1.43	0.53
Thbs1	1.29	0.92	0.62	0.57	1.48	0.72	1.22	0.92
Thbs2	2.18	0.17	1.02	0.75	1.71	0.08	1.67	0.16
Thbs3	2.13	0.56	0.8	0.79	1.86	0.76	2.57	0.33
Vcam1	1.52	0.50	0.71	0.57	1.35	0.71	1.61	0.39

Supplementary Table S3: Fold change data of ECM protease and inhibitors post laser (significant changes in bold)

	**Day 7**		**D21**		**D35**		**D49**	
**Gene Symbol**	**Fold Change**	**P-Value**	**Fold Change**	**P-Value**	**Fold Change**	**P-Value**	**Fold Change**	**P-Value**
Adamts1	2	0.23	0.99	0.95	1.6	0.41	1.39	0.66
Adamts2	1.44	0.40	0.73	0.43	0.97	0.85	1	0.88
Adamts5	1.61	0.31	0.93	0.93	1.27	0.52	1.68	0.11
Adamts8	1.5	0.90	0.73	0.56	1.49	0.95	2.17	0.38
Mmp10	0.77	0.08	0.9	0.74	**0.65**	0.01	1.34	0.30
Mmp11	1.67	0.51	0.69	0.90	1.77	0.40	2.34	0.16
Mmp12	0.97	0.85	0.94	0.89	0.88	0.54	1.44	0.36
Mmp13	0.97	0.94	1.22	0.47	0.96	0.99	1.62	0.12
Mmp14	2.05	0.74	0.71	0.66	2.25	0.62	2.65	0.40
Mmp15	2.17	0.74	0.62	0.65	2.03	0.87	2.75	0.45
Mmp1a	1.31	0.79	0.56	0.48	1.23	0.98	1.55	0.54
Mmp2	1.59	0.63	0.64	0.58	1.51	0.73	1.89	0.38
Mmp3	1.72	0.22	0.87	0.20	**0.79**	0.01	1.46	0.09
Mmp7	0.82	0.29	0.81	0.23	**0.65**	0.01	1.07	0.60
Mmp8	**0.75**	0.05	0.9	0.68	**0.65**	0.01	1.32	0.37
Mmp9	1.51	0.87	0.69	0.57	1.45	1.00	1.85	0.61

Timp1	2.06	0.35	0.8	0.73	1.51	0.79	1.91	0.41
Timp2	1.86	0.51	0.87	0.82	1.84	0.52	2.3	0.25
Timp3	1.34	0.68	0.8	0.82	1.55	0.45	1.54	0.46

Supplementary Table S4: Fold change of other ECM proteins post laser (significant changes in bold)

	**Day 7**		**D21**		**D35**		**D49**	
**Gene Symbol**	**Fold Change**	**P-Value**	**Fold Change**	**P-Value**	**Fold Change**	**P-Value**	**Fold Change**	**P-Value**
Entpd1	1.77	0.55	0.78	0.78	1.26	0.97	2.02	0.36
Hc	0.87	0.67	0.81	0.23	**0.65**	0.01	1.14	0.11
Tgfbi	3.46	0.06	1.7	0.37	2.27	0.11	1.97	0.20

Supplementary Table S5: Fold change of ECM components PFD samples (significant changes in bold)

	**Day 21 PFD**		**D35 PFD**		**D42 PFD**		**D42 PFD D35**	
**Gene Symbol**	**Fold Change**	**P-Value**	**Fold Change**	**P-Value**	**Fold Change**	**P-Value**	**Fold Change**	**P-Value**
Cd44	1.17	0.98	1.13	0.90	1.27	0.83	0.98	0.66
Cdh1	0.81	0.70	0.81	0.63	0.98	0.83	0.67	0.17
Cdh2	1.68	0.54	1.81	0.62	1.36	0.40	1.31	0.38
Cdh3	0.67	0.28	0.66	0.26	0.5	0.13	0.45	0.10
Cdh4	1.1	0.50	1.28	0.63	1.13	0.52	1.09	0.49
Cntn1	0.73	0.30	0.83	0.46	0.79	0.37	0.66	0.25
Ctnna1	0.94	0.49	1.03	0.63	0.98	0.54	0.8	0.37
Ctnna2	0.79	0.37	**0.89**	0.50	0.81	0.39	0.74	0.33
Ctnnb1	0.89	0.48	1.03	0.70	0.81	0.40	0.75	0.34
Icam1	2.64	0.09	**2.8**	0.05	1.96	0.29	2.3	0.14
Itga2	1.13	0.86	1.07	0.98	1.12	0.91	1.13	0.87
Itga3	0.7	0.27	0.79	0.36	0.74	0.30	0.69	0.24

Itga4	0.64	0.15	0.78	0.30	0.8	0.35	0.82	0.37
Itga5	1.19	0.52	1.48	0.76	1.37	0.64	1	0.39
Itgae	2	0.20	27.33	0.37	1.84	0.26	1.9	0.21
Itgal	1.23	0.93	1.43	0.81	1.19	0.90	1.04	0.73
Itgam	1.09	0.90	1.04	0.75	0.97	0.61	0.9	0.51
Itgav	0.77	0.32	0.82	0.42	0.82	0.39	0.72	0.26
Itgax	0.87	0.57	0.72	0.38	0.59	0.18	0.66	0.27
Itgb1	0.82	0.42	0.91	0.55	0.84	0.43	0.82	0.41
Itgb2	**2.24**	0.05	2.34	0.06	**2.38**	0.02	**2.27**	0.02
Itgb3	**4.5**	0.03	3.98	0.06	**4.02**	0.02	**3.77**	0.03
Itgb4	1.13	0.50	1.27	0.54	1.35	0.61	1.13	0.45
Ncam1	1.13	0.62	1.32	0.83	1.19	0.67	1.11	0.58
Ncam2	0.59	0.16	0.77	0.34	0.7	0.24	0.67	0.21
Pecam1	0.82	0.39	0.88	0.43	0.76	0.34	0.84	0.40
Postn	0.44	0.13	0.54	0.17	0.45	0.13	0.43	0.12
Sele	**3.66**	0.02	**3.71**	0.05	**3.49**	0.01	**3.23**	0.00
Sell	2.2	0.07	2.46	0.09	**2.29**	0.01	**2.38**	0.04
Selp	3.93	0.01	**3.27**	0.01	**3.35**	0.02	**2.76**	0.01
Sgce	**0.62**	0.22	0.73	0.35	0.61	0.21	0.56	0.18
Thbs1	1.04	0.61	0.83	0.40	0.83	0.39	0.66	0.27
Thbs2	1	0.86	0.94	0.65	0.74	0.29	0.94	0.64
Thbs3	1.56	0.91	1.7	0.96	1.51	0.85	1.51	0.85

Supplementary Table S6: Fold change of Transmembrane and adhesion molecules PFD samples (significant changes in bold)

	Day 21 PFD		D35 PFD		D42 PFD		D42 PFD D35	
**Gene Symbol**	**Fold Change**	**P-Value**	**Fold Change**	**P-Value**	**Fold Change**	**P-Value**	**Fold Change**	**P-Value**
Col1a1	1.88	0.66	1.78	0.75	1.81	0.74	1.75	0.81
Col2a1	1.35	0.87	1.52	0.61	1.21	0.91	1.02	0.64
Col3a1	0.39	0.04	**0.29**	0.02	**0.32**	0.02	**0.28**	0.02
Col4a1	**0.4**	0.0001	**0.42**	0.0010	**0.39**	0.0001	**0.35**	0.0001
Col4a2	0.48	0.17	0.66	0.26	0.57	0.21	0.54	0.19
Col4a3	1.92	0.12	2.03	0.10	**2.47**	0.05	**2.74**	0.01
Col5a1	1.11	0.55	1.34	0.78	1.18	0.61	1.08	0.52
Col6a1	1.15	0.59	1.28	0.71	1.06	0.51	1.05	0.50
Ecm1	1.15	0.71	1.17	0.75	1.02	0.56	0.94	0.48
Emilin1	1.5	0.86	1.46	0.86	1.46	0.82	1.51	0.87
Fn1	0.94	0.44	1.04	0.55	0.87	0.38	0.77	0.32
Hapln1	0.61	0.28	0.84	0.48	0.7	0.32	0.67	0.29
Lama1	1.01	0.48	1.13	0.60	0.84	0.37	0.89	0.39
Lama2	0.5	0.09	0.52	0.07	**0.43**	0.05	0.52	0.06
Lama3	0.87	0.50	1.03	0.84	0.8	0.38	0.87	0.50
Lamb2	1.35	0.79	1.4	0.72	1.14	0.86	1.07	0.73
Lamb3	2.81	0.10	2.46	0.16	2.11	0.27	2.1	0.26
Lamc1	1.47	0.66	1.65	0.78	1.42	0.61	1.33	0.55
Sparc	0.74	0.32	0.73	0.35	0.57	0.23	0.54	0.21
Spock1	0.68	0.28	0.85	0.44	0.78	0.35	0.69	0.29
Syt1	0.87	0.46	1.09	0.73	1.04	0.66	1.02	0.62
Tnc	1.03	0.42	1.38	0.69	1.14	0.47	0.89	0.33
Vcan	0.88	0.98	0.65	0.06	0.75	0.88	0.87	0.99
Vtn	1.07	0.73	1.17	0.97	0.9	0.51	1.62	0.47

Supplementary Table S7: Fold change of ECM protease and inhibitors PFD samples (significant changes in bold)

	Day 21 PFD		D35 PFD		D42 PFD		D42 PFD D35	
**Gene Symbol**	**Fold Change**	**P-Value**	**Fold Change**	**P-Value**	**Fold Change**	**P-Value**	**Fold Change**	**P-Value**
Adamts1	0.87	0.46	1.11	0.82	0.81	0.41	0.67	0.27
Adamts2	1.36	0.51	1.17	0.84	1.34	0.50	0.96	0.68
Adamts5	0.88	0.51	0.92	0.61	0.8	0.36	0.75	0.29
Adamts8	1.95	0.55	2.28	0.31	2.07	0.46	1.77	0.72
Mmp10	1.51	0.30	**2.25**	0.04	1.52	0.22	1.51	0.24
Mmp11	1.18	0.83	1.11	0.70	1.18	0.81	1.01	0.57
Mmp12	2.07	0.10	**2.16**	0.05	2.18	0.06	**2.09**	0.04
Mmp13	1.6	0.11	1.83	0.17	1.28	0.33	1.32	0.26
Mmp14	0.78	0.28	0.83	0.31	0.93	0.34	0.64	0.23
Mmp15	1.21	0.48	1.37	0.56	1.32	0.53	1.07	0.39
Mmp1a	0.71	0.31	0.92	0.51	0.87	0.46	0.82	0.40
Mmp2	1.09	0.65	1.15	0.74	1.12	0.67	0.88	0.43
Mmp3	1.1	0.84	0.88	0.77	0.63	0.25	0.6	0.24
Mmp7	1.98	0.11	**2.43**	0.03	1.67	0.15	**1.92**	0.05
Mmp8	**3.17**	0.04	**3.56**	0.03	**3.26**	0.00	**3.03**	0.01
Mmp9	0.82	0.36	1.12	0.59	0.94	0.43	0.87	0.38
Timp1	2.69	0.20	3.16	0.10	3.07	0.10	2.4	0.27
Timp2	0.97	0.50	1.12	0.65	0.95	0.47	0.93	0.46
Timp3	0.81	0.39	0.76	0.38	0.76	0.34	0.67	0.27

Supplementary Table S8: Fold change of other ECM proteins PFD samples (significant changes in bold)

	**Day 21 PFD**		**D35 PFD**		**D42 PFD**		**D42 PFD D35**	
**Gene Symbol**	**Fold Change**	**P-Value**	**Fold Change**	**P-Value**	**Fold Change**	**P-Value**	**Fold Change**	**P-Value**
Ctgf	0.55	0.14	0.86	0.49	0.6	0.15	0.64	0.18
Entpd1	1.69	0.66	1.67	0.69	1.69	0.67	1.53	0.86
Hc	1.02	0.96	1.26	0.57	1.04	0.98	1.01	0.86
Tgfbi	**2.9**	0.04	**3.16**	0.04	**3.01**	0.02	**2.7**	0.03
